# Calmodulin Enhances Cryptochrome Binding to INAD in *Drosophila* Photoreceptors

**DOI:** 10.3389/fnmol.2018.00280

**Published:** 2018-08-20

**Authors:** Gabriella Margherita Mazzotta, Massimo Bellanda, Giovanni Minervini, Milena Damulewicz, Paola Cusumano, Simona Aufiero, Monica Stefani, Barbara Zambelli, Stefano Mammi, Rodolfo Costa, Silvio C. E. Tosatto

**Affiliations:** ^1^Department of Biology, University of Padova, Padova, Italy; ^2^Department of Chemical Sciences, University of Padova, Padova, Italy; ^3^Department of Biomedical Sciences, University of Padova, Padova, Italy; ^4^Department of Cell Biology and Imaging, Institute of Zoology and Biomedical Research, Faculty of Biology and Earth Sciences, Jagiellonian University, Kraków, Poland; ^5^Department of Pharmacy and Biotechnology, University of Bologna, Bologna, Italy; ^6^CNR Institute of Neuroscience, Padova, Italy

**Keywords:** *Drosophila melanogaster*, calmodulin, INAD, cryptochrome, photoreception

## Abstract

Light is the main environmental stimulus that synchronizes the endogenous timekeeping systems in most terrestrial organisms. *Drosophila* cryptochrome (dCRY) is a light-responsive flavoprotein that detects changes in light intensity and wavelength around dawn and dusk. We have previously shown that dCRY acts through Inactivation No Afterpotential D (INAD) in a light-dependent manner on the Signalplex, a multiprotein complex that includes visual-signaling molecules, suggesting a role for dCRY in fly vision. Here, we predict and demonstrate a novel Ca^2+^-dependent interaction between dCRY and calmodulin (CaM). Through yeast two hybrid, coimmunoprecipitation (Co-IP), nuclear magnetic resonance (NMR) and calorimetric analyses we were able to identify and characterize a CaM binding motif in the dCRY C-terminus. Similarly, we also detailed the CaM binding site of the scaffold protein INAD and demonstrated that CaM bridges dCRY and INAD to form a ternary complex *in vivo*. Our results suggest a process whereby a rapid dCRY light response stimulates an interaction with INAD, which can be further consolidated by a novel mechanism regulated by CaM.

## Introduction

Endogenous timekeeping systems allow living organisms to synchronize their behavior and physiology to daily and seasonal changes of the environment. Despite being self-sustained, the oscillatory mechanism that generates such rhythmicity needs to be entrained by environmental stimuli such as light, temperature, food and social interactions. In the majority of instances, light is the predominant entrainment cue. Most organisms use changes in features and intensity of light around dawn and dusk as their primary Zeitgeber (Roenneberg and Foster, 1997). In *Drosophila melanogaster*, the blue-light resetting of the circadian clock mainly relies on the action of CRYPTOCHROME (dCRY). Upon light activation, dCRY binds to the clock protein TIMELESS (TIM) and the ubiquitin ligase JETLAG, promoting degradation of both TIM and itself (Busza et al., 2004; Koh et al., 2006; Peschel et al., 2009; Ozturk et al., 2011). Like other members of the conserved blue-light absorbing flavoprotein family, dCRY possesses an N-terminal light-sensing domain and a small C-terminal tail. Following illumination, the C-terminus is released from the surface of the N-terminal domain onto which it is folded, allowing partner proteins to bind dCRY in a light dependent manner (Ozturk et al., 2011). We have previously demonstrated an important role for the dCRY C-terminus in mediating molecular interactions. This terminus harbors several protein-protein interaction motifs involved in the function and regulation of this complex protein, including a TRAF2 ligand and two class III PDZ-binding motifs (Hemsley et al., 2007; Mazzotta et al., 2013). PDZ (postsynaptic density protein 95, *Drosophila* disk large tumor suppressor, and zonula occludens-1 protein) domains assemble large protein complexes involved in signaling processes (Ivarsson, 2012) by binding a number of different short linear motifs frequently localized at the C-termini of proteins (Stein et al., 2009).

One of the most studied model systems for the role of scaffolds in signal transduction is Inactivation No Afterpotential D (INAD), a five PDZ containing protein in the microvilli of *Drosophila* photoreceptor cells. INAD organizes the core components of the phototransduction pathway into a supramolecular complex (Montell, 1998, 2012; Huber, 2001). The assembly of the INAD signaling complex is highly coordinated and spatio-temporally organized. Signaling component binding involves specific INAD regions, usually more than one PDZ domain, and multiple targets binding to the same PDZ domain are not infrequent (Liu et al., 2011). INAD is also able to form homodimers, increasing the ability of the complex to simultaneously link multiple targets (Xu et al., 1998). This specific interaction involves the dCRY C-terminus and a specific INAD region comprising the PDZ2-PDZ3 tandem (Mazzotta et al., 2013). The binding of dCRY to INAD has connected this circadian photoreceptor with the visual transduction complex, where it modulates visual responses, measured as both photoreceptor sensitivity and motion vision, in a circadian fashion (Mazzotta et al., 2013).

Several aspects of the visual response in both vertebrates and invertebrates are regulated by a Ca^2+^/calmodulin (CaM) dependent signaling mechanism. In *D. melanogaster*, for example, light-stimulated rhodopsin initiates a phospholipase C signaling cascade resulting in opening of the Transient Receptor Potential (TRP) and TRPL (TRP-like) cation channels, leading to Na^+^ and Ca^2+^ influx (Montell, 2012). Rhabdomeres in the fly retina, microvillar photoreceptor cell structures, contain high levels of CaM. Several CaM targets have been identified in the signaling cascade, corroborating the critical role of Ca^2+^. CaM binds the eye-specific kinase neither inactivation nor afterpotential C (NINAC), an unconventional myosin responsible for rhabdomeric localization (Porter et al., 1993). Both light-sensitive TRP and TRPL channels possess CaM binding sites (one in TRP and two in TRPL) and bind CaM *in vitro* (Phillips et al., 1992; Warr and Kelly, 1996). The rhodopsin phosphatase Retinal degeneration C (RdgC) also binds CaM, and this interaction is important for photoresponse termination (Lee and Montell, 2001). CaM interaction regulates the activity of Ca^2+^/CaM dependent kinase II (CaMKII), which is abundant in fly retina and involved in the negative regulation of visual responses (Lu et al., 2009). A direct interaction is also reported between CaM and INAD, involving the region upstream of the PDZ2 domain of the latter (Chevesich et al., 1997; Tsunoda et al., 1997; Xu et al., 1998). Given the scaffold nature of INAD, it can be assumed that the interaction with CaM may both promote and regulate INAD binding to different partners, e.g., dCRY. CaM is an ubiquitous sensor protein of 148 amino acids containing two domains connected by a flexible linker (Clapham, 2007) and is extremely conserved from yeast to human. Each domain hosts two “EF-hands,” helix-loop-helix motifs binding Ca^2+^ with varying affinity, often modulated by the interaction with target proteins (Clapham, 2007). Upon Ca^2+^ binding, each CaM domain undergoes a conformational change. CaM recognizes target proteins through a “CaM-binding-domain” (CaMBD). Generally, the binding region on the target protein is a stretch of about 20 amino acids, with high hydrophobic content and a tendency to form α-helices. CaMBD binding is largely driven by hydrophobic interactions between “anchor” residues on the target and methionine side chains in the CaM pocket, which become exposed upon Ca^2+^ binding (Yamniuk et al., 2007; Marshall et al., 2015). The binding mechanism can be very diverse, resulting in different tuning of target protein properties. The two CaM domains can interact with the same CaMBD (typical “wrap around” mode) or the N- and C- lobe may bind the different domains independently. In the latter case, CaM acts as an adaptor protein: the binding can promote structural reorganization if the two target domains are on the same protein, or induce dimerization when different proteins are involved (Yamniuk et al., 2007). Here, we present a novel interaction between dCRY and CaM. Through *in silico* analysis, *in vitro* assays and *in vivo* experiments, we identified the CaM binding motif in the dCRY N-terminus precisely. We also characterized a CaM binding site in the INAD stretch upstream of PDZ2, and demonstrated that dCRY, INAD and CaM form a complex *in vivo*. Our data suggest a role for CaM as a novel regulatory module in the formation of the dCRY/INAD complex that strengthens light-induced responses.

## Materials and Methods

### *In silico* Analyses

UniProt (The UniProt Consortium, 2016) canonical sequences (UniProt accession numbers in parentheses) for dCRY (O77059) and INAD (Q24008) were retrieved. Alignment was performed with T-Coffee (Notredame et al., 2000) using default parameters and visualized with Jalview (Waterhouse et al., 2009). Secondary structure and sequence features were predicted with FELLS (Piovesan et al., 2017). CaM binding sites were predicted with the CaM target database (Yap et al., 2000). A protein-protein interaction network centered on dCRY, INAD and CaM was derived from STRING (Szklarczyk et al., 2017) with no more than 20 interactors for the second shell, and a default interaction score confidence parameter of 0.400. Nuclear magnetic resonance (NMR) guided 3D-structure prediction was performed with CS-Rosetta (Shen et al., 2009) paired with Rosetta 3.8 (Leaver-Fay et al., 2011), using default settings and protocols.

### Calmodulin Expression and Purification

*Escherichia coli* BL21 cells transformed with pET28 encoding (His)_6_-CaM (kindly provided by Prof. Giuseppe Zanotti, Department of Biomedical Sciences, University of Padova) were grown at 37°C and 220 rpm in M9 minimal medium containing 4.4 g/L ^13^C-glucose monohydrate and/or 1 g/L ^15^NH_4_Cl and 50 μg/mL kanamycin. Recombinant protein expression was induced at OD600 ~0.6 with 1 mM isopropyl-β-D-thiogalactopyranoside (IPTG) overnight at 37°C and 200 rpm, and twice harvested by centrifugation (5,000 rpm, 25 min, 4°C). The cell pellets were resuspended in 20 mL of 50 mM Tris-HCl buffer pH 6.5 with protease inhibitors (Complete Protease Inhibitor Cocktail Tablets, Roche, Basel, CH) and lysozyme, and disrupted by sonication. The lysate was centrifuged at 18,000 rpm for 45 min at 4°C and the supernatant filtered with a 0.45 μm filter, before loading it into a Phenyl Sepharose resin. The resin was mechanically stirred at 4°C for 1 h and then centrifuged at 1,000 rpm for 15 min at 4°C. The supernatant was filtered with a 0.45 filter and loaded into a series of two Phenyl Sepharose HP, 5 mL columns (GE Healthcare, Chicago, IL, USA) equilibrated with 50 mM Tris-HCl buffer, 1 mM CaCl_2_, pH 6.5. The columns were washed with 50 mM Tris-HCl buffer, 1 mM CaCl_2_, 100 mM NaCl, pH 6.5 and the protein eluted with 50 mM Tris-HCl buffer, 1 mM EGTA, pH 6.5. Fractions containing the protein were loaded on a Hi-Prep^®^ desalting column (GE Healthcare, Chicago, IL, USA) and eluted with 50 mM phosphate buffer at pH 6.5. This protocol yielded 30÷40 mg of protein per liter of culture in M9. For the NMR experiments, the buffer was supplemented with 5 mM CaCl_2_ and the protein was concentrated up to 1.2 mM by ultrafiltration (Vivaspin, 5 kDa MWCO).

### Peptide Synthesis

The peptide corresponding to INAD_230–243_ was prepared by stepwise solid-phase synthesis using Fmoc strategy on a MultiSynTech semi-automated peptide synthesizer. Fmoc-amino acids and rink amide MBHA resin were purchased from Iris Biotech GmbH (Marktredwitz, DE). Cleavage reactions were performed in a TFA/TIS/H_2_O mixture (95:2.5:2.5) for 1 h at room temperature. The crude peptide was purified by accelerated chromatographic isolation (IsoleraTM Spektra, Biotage, Uppsala, S). Eluted fractions were verified by analytical HPLC and ESI mass spectrometry and lyophilized. The dCRY_490–516_ peptide (purity >99%) was purchased from ThermoFisher Scientific (Waltham, MA, USA). Both peptides were acetylated at the N-terminus and amidated at the C-terminus, to mimic the protein environment and remove extra charges.

### Nuclear Magnetic Resonance (NMR) Experiments

All NMR experiments were performed with a Bruker DMX 600 MHz spectrometer with a room temperature probe, at 303 K. ^15^N-HSQC experiments were collected with eight scans, 2048 complex data points and a spectral width of 14 ppm in the ^1^H dimension, and 200 increments and a spectral width of 25 ppm in the ^15^N dimension. Samples of 340–380 μM uniformly ^15^N-labeled CaM were titrated with peptide stock solutions (3.2 mM for INAD_230–243_ and 2.8 mM for dCRY_490–516_). The protein and the peptide were dissolved in the same buffer consisting of 50 mM Tris-Cl, 100 mM NaCl, 5 mM CaCl_2_ at pH 6.5. The pH of the solutions was checked and adjusted after dissolving CaM and the peptides, to avoid pH changes during the titration. Deuterated water (10% v/v) was added to the NMR tube. Resonance assignment of the ^15^N-HSQC for the Ca^2+^ loaded apo-CaM spectrum was achieved by comparison with data available in the BMRB database^1^ and confirmed by HNCA and HNCACB 3D heteronuclear experiments, using a sample of 1 mM protein in the same buffer described above. Amide chemical shift perturbations were calculated as $\sqrt[{2}]{\frac{{{(}^{1}H\Delta \delta )}^{2}+{\left(0.14\times^{15}N\Delta \delta \right)}^{2}}{2}}$ where ^1^HΔδ and ^15^NΔδ are the ^1^H and ^15^N amide chemical shift changes, respectively. Data were processed with TOPSPIN 3.1 (Bruker BioSpin GmbH, Rheinstetten, Germany) and analyzed using CARA 1.9 Keller, 2004 and Sparky (Lee et al., 2015).

## Yeast Two-Hybrid Assays

The experiments were performed in the EGY48 yeast strain (MATα, *ura3*, *trp1*, *his3*, 3LexA-operator-LEU). Baits were prepared by cloning the sequence of interest fused to the LexA moiety in the bait vector (pEG202), while preys contained the desired proteins fused to the “acid-blob” portion of the prey vector (pJG4–5; Golemis and Brent, 1997). The *Xenopus laevis CaM* (xCaM) sequence (full length and fragments) was amplified from the pET28 encoding (His)_6_-CaM described above and used either as bait or as prey. The primers used are listed in Supplementary Table S1. All clonings were performed by using the In-Fusion^®^ HD Cloning Kit (Clontech, Mountain View, CA, USA). The constructs were fully sequenced to assess the in-frame insertion of the cDNA and to control for unwanted mutations. Quantification of β-galactosidase activity was performed in liquid culture as in Ausbel (1998). A Student *t*-test was used to perform single group comparisons.

## Fly Strains

The following strains of *Drosophila melanogaster* were used: *yw;tim*-GAL4 (Emery et al., 1998), *UAS-HAcry* (Dissel et al., 2004). Flies were maintained on a standard cornmeal medium under LD 12:12 regime at constant 23°C.

### CoIP and Western-Blot

Head extracts from overexpressing HACRY flies raised in 12:12 light:dark cycles and collected at Zeitgeber Time 24 (ZT24), before lights on, and after a 15-min light pulse (Mazzotta et al., 2013), were subjected to coimmunoprecipitation (CoIP) by using an anti-high affinity (anti-HA) Affinity Matrix (Roche, Basel, Switzerland), following manufacturer instructions. SDS PAGE was performed as previously described (Mazzotta et al., 2013) and immunocomplexes were analyzed using the following antibodies: rabbit polyclonal anti-INAD (1:500; Wes et al., 1999), rabbit monoclonal [EP799Y] anti-CaM (1:1,000; AbCam, Cambridge, UK) and mouse anti-HA (1:5,000; Sigma Aldrich, St. Louis. MO, USA). An anti-rabbit IgG HRP (1:3,000; BioRad Laboratories, Hercules, CA, USA) and an anti-mouse IgG HRP (1:5,000; Sigma Aldrich, St. Louis. MO, USA) were used as secondary antibodies. For quantification of the immunodetected signals, films were analyzed with ImageJ software (available at http://rsb.info.nih.gov/ij, developed by Wayne Rasband, National Institutes of Health). Relative abundance of CaM was defined as a ratio with HACRY (CaM/HACRY).

### CaM Pulldown Assay

*Drosophila* S2R+ cells (Invitrogen), maintained at 25°C in Schneider’s *Drosophila* medium (Thermo Scientific), were transfected with 1 μg of pAc-HACRY (a gift form Ezio Rosato), using the Effectene Transfection Reagent (QIAGEN), following manufacturer instruction. Seventy-two hours after transfection, proteins were extracted in TritonX-100 lysis buffer (20 mM Hepes pH 7.5; 100 mM KCl; 5% glycerol; 0.5% TritonX-100; 1 mM DTT; 1× Complete Protease Inhibitor Cocktail-Roche). Protein extracts were subjected to CaM binding assay using the CaM-Sepharose Beads (BioVision, Inc.). The extract was divided equally in two conditions and CaCl_2_ or EDTA were added up to 2 mM and 5 mM concentration, respectively. 100 μL of beads washed with H_2_O and riequilibrated with Lysis Buffer were added and samples were incubated at 4°C for 2 h on a rotating wheel. Samples were centrifuged at 1,500 rcf for 1 min. The supernatant (containing unbound protein) was carefully removed and the beads were washed three times using 1 mL of Lysis buffer. CaM bound proteins were detached from the beads by the addition of loading buffer (LDS—Invitrogen^®^) and heating at 70°C for 10 min and analyzed by SDS-PAGE on 4%–12% NuPAGE^®^ Novex^®^ Bis-Tris Gels (Thermo Fisher). Western Blot was performed with monoclonal anti-HA primary antibody (1:2,000; Sigma Aldrich, St. Louis. MO, USA) and anti-mouse IgG HRP secondary antibody (1:5,000; Sigma Aldrich, St. Louis. MO, USA).

### Isothermal Titration Calorimetry (ITC)

Peptide titrations were performed at 25°C using a high-sensitivity VP-isothermal titration calorimetry (ITC) microcalorimeter (MicroCal LLC, Northampton, MA, USA). The reference cell was filled with deionized water. Protein and peptide solutions were prepared by diluting concentrated stock solutions in the reaction buffer (50 mM TrisHCl, pH 7.5, 150 mM NaCl, in the absence or in the presence of 5 mM CaCl_2_). Apo-protein and peptide solutions were devoid of Ca^2+^ ions, as determined by inductively coupled plasma emission spectroscopy, as previously described (Merloni et al., 2014). Each experiment started with a small injection of 1–2 μL, which was discarded from the analysis of the integrated data, in order to avoid artifacts due to the diffusion through the injection port occurring during the long equilibration period, locally affecting the protein concentration near the syringe needle tip. Care was taken to start the first addition after baseline stability had been achieved. In each individual titration, small volumes (5–10 μL) of a 0.4–0.8 mM solution containing dCRY or INAD peptide was injected into a solution of 20–40 μM CaM, using a computer-controlled 310-μL microsyringe. To allow the system to reach equilibrium, a spacing of 300 s was applied between each ligand injection. Competition experiments were performed in the presence of 5 mM CaCl_2_, by titrating dCRY peptide (800 μM) over a solution containing CaM (40 μM) and INAD peptide (160 μM), or by titrating INAD peptide (800 μM) over a solution containing CaM (40 μM) and dCRY peptide (40 μM).

Integrated heat data obtained for each titration were fitted using a nonlinear least-squares minimization algorithm to a theoretical titration curve, using AFFINImeter^2^, using both the independent sites and the stoichiometric equilibria approach. *ΔH* (reaction enthalpy change, cal mol^−1^) and *K*_a_ (binding constant, M^−1^) were the thermodynamic fitting parameters. The parameters *r*_M_ (scaling parameter for the protein concentration) and Q_dil_ (heat of dilution, cal mol^−1^) were also adjusted as fitting parameters. The reaction entropy was calculated using the relationships *ΔG* = −*RT*ln*K*_a_ (*R* = 1.9872 cal mol^−1^ K^−1^, *T* = 298 K) and *ΔG* = *ΔH* − *TΔS*. A global fitting analysis was performed for the curves representing the titrations of each peptide into the CaM solution and the titration of dCRY into the CaM-INAD complex. The reliability of the obtained fits was evaluated using the Goodness of Fit (GoF) parameter.

## Results

In 2013, we documented an interaction between dCRY and the visual cascade components thorough the scaffold protein INAD, and we showed this interaction to be of functional importance in fly vision (Mazzotta et al., 2013). We also established that the dCRY-INAD interaction is mediated by a specific INAD region, comprising the PDZ2-PDZ3 tandem extended upstream of PDZ2, including an amino acid stretch (i.e., ^233^TMAKINKR^240^) known to be part of a CaM binding motif (Xu et al., 1998; Mazzotta et al., 2013). In the fly retina, CaM is concentrated in rhabdomeres, photoreceptor cell microvillar structures where the phototransduction cascade complex (Signalplex), assembled by the INAD scaffold protein, is localized (Shieh and Niemeyer, 1995; Huber et al., 1996; Shieh and Zhu, 1996; Chevesich et al., 1997; Tsunoda et al., 1997). dCRY is another member of the Signalplex, in which it interacts with the phototransduction complex through INAD, and contributes to the fly circadian visual response (Mazzotta et al., 2013). This interaction is mainly driven by light exposure, suggesting that other modulators can mediate the interaction in a light independent fashion. To shed light on a potential, wider role for CaM in clock entrainment, an interaction network centered on dCRY, INAD and CaM was generated with STRING (Szklarczyk et al., 2017). The resulting network (Supplementary Figure S1) shows that proteins forming the Signalplex are physically linked to the circadian timekeeping mechanism through dCRY. CaM interacts with multiple components of the Signalplex, i.e., NinaC, TRP, TRPL and INAD, presumably acting as both driver and mediator of different cell signals (Supplementary Figure S1). The connection between INAD and Galphaq (G protein alpha q subunit) is also of interest. This protein is actively expressed in chemosensory cells and central neurons (Talluri et al., 1995) and is known to be required for correct signal phototransduction in *D. melanogaster* (Lee et al., 1990). Based on these findings, we hypothesized that CaM associates the complex formed by dCRY with INAD, serving as a direct molecular bridge between light sensing and signal propagation.

### CaM Interacts With the Circadian Blue-Light Photoreceptor dCRY

**Figure 1:**
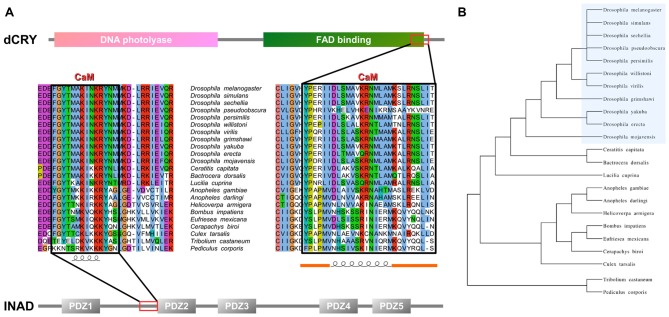
Inactivation No Afterpotential D (INAD) and *drosophila* cryptochrome (dCRY) sequence domain organization and calmodulin (CaM) binding site conservation. **(A)** PDZ domains composing INAD are presented in gray bars named with the corresponding Pfam nomenclature, while the CaM binding site is boxed in red. Sequence conservation shows the region partially conserved among different species. Secondary structure prediction is presented below. Domains composing dCRY are presented as colored bars and named with the corresponding Pfam nomenclature, while the CaM binding site is boxed in red. Sequence conservation shows the region partially conserved among different species. Secondary structure prediction is presented below, with orange bars representing intrinsically disordered segments. **(B)** Evolutive tree representing the distance among organisms used to perform the conservation analysis.

**Figure 2:**
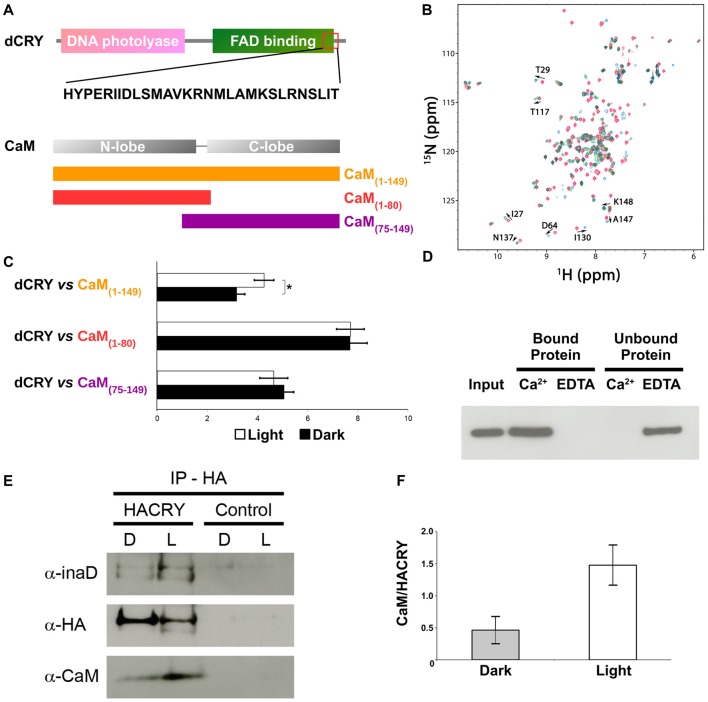
CaM interacts with dCRY. **(A)** CaM binding site in dCRY. **(B)** Superposition of CaM ^15^N-HSQC spectra acquired at increasing peptide:CaM ratio for the peptide dCRY_490–516_ (0:1 in blue, 1:1 in green, 2:1 in red); A subset of assigned peaks exhibiting large shifts upon peptide binding are indicated with arrows. **(C)** Yeast two-hybrid assay in which full-length dCRY (bait) was challenged with CaM (full-length or fragments; prey). As negative control, full-length dCRY was challenged with the empty prey vector, and the measured activity, considered background, was subtracted from that of the samples. The mean ± SEM of seven independent clones, three replicates, are reported. *p < 0.05. **(D)** CaM pulldown assay and western blot showing that dCRY binds CaM in a Ca^2+^ dependent manner. Protein extract from *Drosophila* S2R+ cells overexpressing HACRY (*input*) was divided equally in two parts and the appropriate reagents were added (CaCl_2_ or EDTA). Each extract (containing either CaCl_2_ or EDTA) was incubated with CaM agarose beads to allow binding: *unbound* proteins were removed and the *bound* proteins were detached from the beads. Membrane was probed with an anti-HA antibody. **(E)** Coimmunoprecipitation (CoIP) and western blot confirming the interaction between CRY, INAD and CaM in HACRY-overexpressing flies (HACRY, *yw*;*tim-GAL4*/+; UAS-*HAcry*/+). *yw;tim-GAL4* flies were used as control. Flies were reared in 12:12 light:dark and collected in dark (Zeitgeber Time 24, ZT24, end of dark phase) and light (ZT24 +15-min light pulse). Membranes were probed with anti-CaM, anti-INAD and anti-HA antibodies. **(F)** The affinity of CaM for dCRY at ZT24 (Dark) and after 15 min of light was quantified as the CaM/HACRY ratio. Mean levels of three independent replicates are shown. The difference between CaM/HACRY ratios under light and dark conditions were significant (*p* < 0.02, Student *t*-test).

An analysis of dCRY with the CaM binding database (Yap et al., 2000) suggested a putative binding site in the dCRY C-terminal tail (residues 490–516). This region is predicted to form a short α-helix, a common feature shared among CaM binding regions (Lee and Zheng, 2010; Figure 1 and Supplementary Figure S2). Notably, this region is also in the proximity of the linear motifs previously found responsible for interaction with the INAD PDZ domains (Mazzotta et al., 2013). A multiple sequence alignment of dCRY orthologs shows this region to be conserved among arthropods. The motif is almost identical in all Drosophilidae, with the exception of *D*. *pseudoobscura*, in which different amino acid substitutions are observed. A comparative investigation of secondary structure highlights a short-conserved helix, suggesting an evolutionarily preserved functional role. To verify these predictions, we tested binding of a synthetic peptide mimicking dCRY residues 490–516 to ^15^N-labeled CaM using ^15^N-HSQC experiments (Figure 2). The position and shape of the peaks in the ^15^N-HSQC map used to follow the titrations with the peptide is very sensitive to the chemical environment of the corresponding amino acids and represents a useful tool to study protein interactions with other molecules. Most of the peaks in the HSQC spectrum were perturbed upon addition of the peptide up to a two-molar excess. Most of the perturbed signals moved very little at the beginning of the titration, becoming weaker and broadening beyond detection before reappearing in a different position during the titration (Supplementary Figure S3), which suggests a slow or slow-to-intermediate exchange regime, typically associated with K_d_ in the nM-μM range. As a consequence, it was not possible to follow several peaks during the titration, and to trace the assignment of the bound protein starting from apo-CaM. A number of isolated, representative, peaks on both lobes moved significantly, similar to what observed for other known CaM binding domains. The available data support the binding of the selected peptide to CaM, although they do not allow the definition of the molecular details and interaction stoichiometry. To address these limitations, the thermodynamics of interaction between CaM and the dCRY-derived peptide was studied by means of ITC.

**Figure 3:**
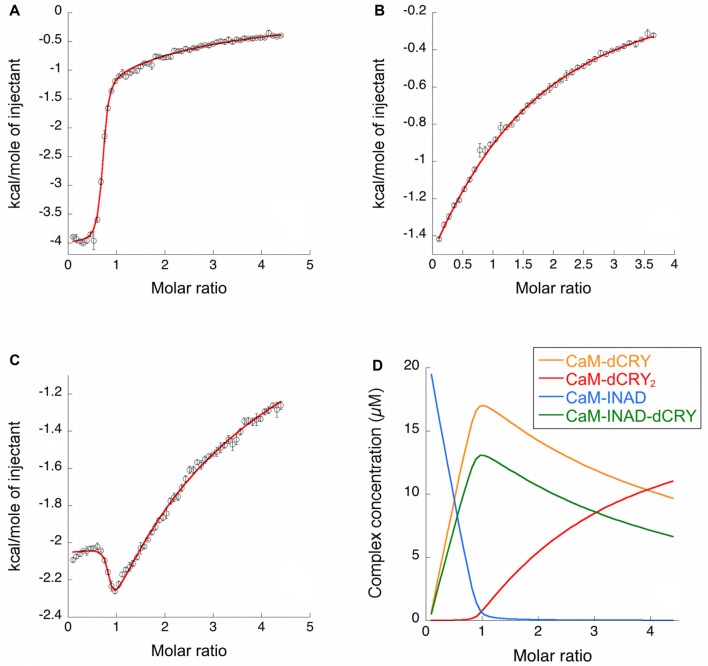
Binding properties of dCRY and INAD to CaM in the presence of 5 mM Ca^2+^ determined using isothermal titration calorimetry (ITC). **(A)** Integrated heat data of dCRY over CaM as a function of peptide/protein molar ratio. The continuous line represents the best fit (goodness of fit, GoF = 66.9%) obtained using a global fit and a model involving binding of two dCRY peptides to the protein (CaM-dCRY_2_), according to Scheme 1. **(B)** Integrated heat data of INAD over CaM as a function of peptide/protein molar ratio. The continuous line represents the best fit (GoF = 71.6%) obtained using a global fit and model involving binding of one INAD peptide to the protein, according to Scheme 3. **(C)** Integrated heat data of dCRY over CaM, in the presence of a four times higher concentration of INAD, as a function of peptide/protein molar ratio. The continuous line represents the best fit (GoF = 64.7%) obtained by modeling the binding isotherm using the binding scheme in Scheme 5 and a global fit approach. **(D)** Concentration distribution of the different CaM complexes occurring upon the competition experiment performed in **(C)**, calculated using the thermodynamic parameters obtained from the fit and the binding model described in Scheme 5.

**Scheme 1:**
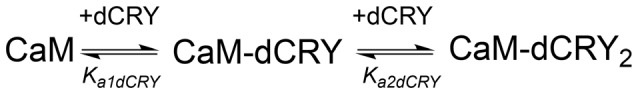
Model describing the stoichiometric binding of two dCRY molecules sequentially interacting with CaM protein, in the presence of Ca^2+^.

The interaction of CaM with dCRY is an exothermic reaction, both in the absence and in the presence of Ca^2+^, as indicated by negative peaks following each injections of peptide into the protein solution (Supplementary Figures S4A,C). The shape of the binding isotherm depends on the presence of Ca^2+^ ions: a curve with two inflection points, indicative of two binding events, is observed with 5 mM Ca^2+^ (Supplementary Figure S4B), while a single event is inferred for the apo-protein (Supplementary Figure S4D). The integrated heat data obtained for dCRY titration into Ca^2+^-CaM (Figure 3A and Supplementary Figure S4B) were fitted using either an approach involving stoichiometric equilibria, considering the stepwise binding of each ligand molecule to the protein (Scheme 1), or a model involving independent ligand-site equilibria, which evaluates the microscopic interaction constant to each binding site. The two binding schemes gave identical results (GoF = 70.65%), indicative of the significant difference in affinity between the two binding events.

**Scheme 2:**
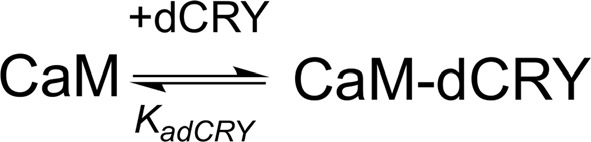
Model describing the binding of a single dCRY molecule to CaM protein, in the absence of Ca^2+^.

The two binding events show affinity constants separated by three orders of magnitude (high affinity (HA) site, *K_a1dCRY_* = 1.71 ± 0.06 × 10^7^ M^−1^, *K*_d1dCRY_ = 58 ± 2 nM; low affinity (LA) site, *K*_a2dCRY_ = 2.31 ± 0.07 × 10^4^ M^−1^, *K*_d2dCRY_ = 43 ± 1 μM). Both events are characterized by favorable enthalpic contributions (Δ*H*_1dCRY_ = −3.73 ± 0.01 kcal mol^−1^ and Δ*H*_2dCRY_ = −4.60 ± 0.09 kcal mol^−1^), and the calculated entropy change is positive (ΔS_1dCRY_ = +20.6 cal mol^−1^ K^−1^ and Δ*S*_2dCRY_ = +4.53 cal mol^−1^ K^−1^). The affinity constants determined by isothermal titration calorimetry (ITC) fully support the behavior of the HSQC peaks observed during the NMR titration. In the absence of Ca^2+^, a single event is apparent and the integrated heat data (Supplementary Figure S4D) could be fitted (GoF = 68.17%) using a scheme involving a single binding site for *drosophila* cryptochrome (dCRY; Scheme 2; *K*_adCRY_ = 3.6 ± 0.1 × 10^5^ M^−1^, *K*_ddCRY_ = 2.78 ± 0.08 μM). Favorable enthalpic and entropic contributions (*ΔH* = −3.26 ± 5 kcal mol^−1^ and *ΔS* = +14.5 cal mol^−1^ K^−1^) are calculated, with values very similar to the ones obtained for the first binding event observed for the Ca^2+^ protein.

On the other hand, the affinity constant is two orders of magnitude lower than the one observed in the presence of Ca^2+^, indicating that Ca^2+^ ions have an important effect in increasing the binding affinity of dCRY to CaM.

### CaM—dCRY Association Is Ca^2+^-Dependent and Strengthened Upon Light Exposure

The ability of CaM to interact with dCRY was tested in a yeast two-hybrid assay using dCRY as bait and CaM as prey. Since CaM consists of two homologous domains with different energetic properties, we decided to test the full-length (CaM_1–149_) protein and either domain separately (CaM_1–80_ and CaM_75–149_). Because of the well-known regulation exerted on dCRY by light even in a cell-based system such as yeast (Rosato et al., 2001; Hemsley et al., 2007; Mazzotta et al., 2013), the interaction was tested both in the light and in the dark. A strong interaction between dCRY and CaM was observed (both full length and fragments). The binding affinity was particularly high when the N-terminal moiety of CaM (aa 1–80) was used as prey (Figure 2). Nevertheless, when the two proteins were tested as full-length [dCRY vs. CaM_1–149_], the interaction in the light was significantly higher than in the dark (*p* < 0.05, Student *t*-test) suggesting that this association may be light-modulated. As dCRY is a well-known light responsive flavoprotein (Masiero et al., 2014), we can speculate that specific structural rearrangements may explain this behavior. On the other hand, light is known to activate a yeast stress-response signaling pathway that leads to an increase in intracellular Ca^2+^ levels (Bodvard et al., 2013), which might cooperatively enhance CaM binding to dCRY. To address this ambiguity, we performed a CaM pulldown assay in the presence or absence of Ca^2+^, to investigate whether or not binding of full-length dCRY to CaM is Ca^2+^-dependent. Protein extracts from *Drosophila* S2R+ cells overexpressing an HA tagged form of dCRY (HACRY) were incubated with CaM sepharose beads in the presence of Ca^2+^ or EDTA (a known Ca^2+^ chelator) and the bound proteins were analyzed by western-blot. The result clearly shows that dCRY binds CaM in a Ca^2+^ dependent manner (Figure 2D).

### dCRY, INAD and Calmodulin Form a Complex *in vivo*

Considering CaM localization in the fly retina, we hypothesized that CaM may also be present in the complex formed by dCRY in fly photoreceptors. Therefore, a CoIP assay was performed, followed by western blot, to test whether dCRY and CaM are also able to interact *in vivo*. Head extracts from transgenic flies overexpressing a hemagglutinin (HA)-tagged form of dCRY (HACRY), raised in 12:12 light:dark cycles collected at ZT24, before lights on or after a 15-min light pulse, were subjected to CoIP with an anti-HA antibody (Mazzotta et al., 2013). The western blot with a CaM specific antibody showed this interaction to be specific and occurring *in vivo* (Figure 2). The same membrane was also probed with antibodies against INAD (Wes et al., 1999) and HA, confirming the previously observed dCRY-INAD interaction. Moreover, the signal corresponding to CaM was more intense in head extracts collected after a light pulse, although in these samples the amount of HACRY was lower because of the well known light-dependent degradation of dCRY (Peschel et al., 2009). Indeed, the difference between CaM/HACRY ratios under light and dark conditions was significant (*p* < 0.02, Student *t*-test; Figure 2F). These results are in line with those of the yeast two-hybrid assays, indicating that CaM, dCRY and INAD form a complex in the fly retina, which is strengthened by light.

### Defining the CaM Binding Site in INAD

**Figure 4:**
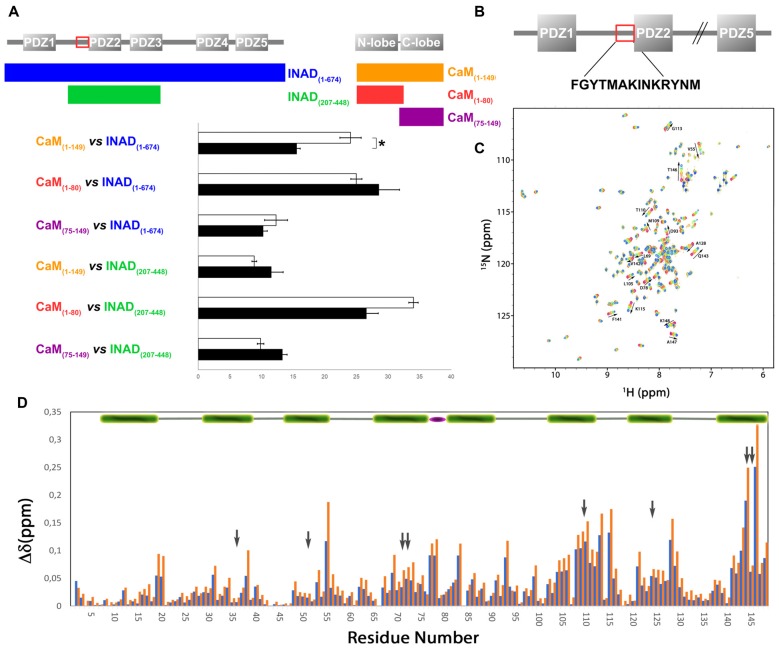
Evidence for an interaction between CaM and INAD. **(A)** CaM (full-length or fragments) was challenged with INAD (full-length or fragment) in a yeast two-hybrid assay with β-galactosidase activity as a measure of interaction. As negative control, CaM was challenged with the empty prey vector and the measured activity, considered as background, was subtracted from that of the samples. The mean ± SEM of seven independent clones, three replicates are reported. *p < 0.001. **(B)** CaM binding site in INAD. **(C)** Superposition of CaM ^15^N-HSQC spectra acquired at increasing peptide:CaM ratio for the peptide INAD_230–243_, i.e., 0:1, 0.3:1, 0.6:1, 1:1, 1.3:1, 1.6:1, 2:1. The direction and magnitude of the chemical shift perturbation of a selected number of CaM amide cross peaks are indicated with arrows. **(D)** Combined chemical shift perturbations of ^15^N-CaM upon addition of one equivalent (blue bars) or two equivalents (red bars) of the peptide INAD_230–243_ as a function of the protein sequence. The gray arrows indicate the position of the eight methionines (four in each domain) that become exposed in the presence of Ca^2+^ and are involved in target sequence binding. The green bar on the top represents helical CaM segments, while the flexible linker between the two domains is represented with a purple ellipse.

In order to verify whether the region immediately upstream PDZ2 does indeed contain a CaM-binding motif, the extended PDZ2-PDZ3 tandem (residues 207–448) was tested for its ability to bind CaM in a yeast two-hybrid assay, challenging CaM (full length or fragments) as bait with INAD as prey. A strong light independent interaction between CaM (both full length and fragments) and this INAD fragment was observed. The binding affinity was particularly high when the N-terminal CaM moiety (residues 1–80) was used as bait (Figure 4). A comparable interaction pattern was observed when CaM was challenged with full-length INAD. As for the CaM/dCRY pair, when the proteins were tested as full length (CaM_1–149_ vs. INAD_1–647_), a statistically significantly stronger affinity in the presence of light was observed (*p* < 0.001, Student *t*-test). The observation that light potentiates the interactions between CaM and both dCRY and INAD supports the hypothesis that a Ca^2+^ flux acts as an interaction enhancer. The data from INAD also suggest that light-induced conformational changes are not relevant for the interaction with CaM. Analysis with FELLS (Piovesan et al., 2017) predicts the ^233^TMAKINKR^240^ motif to form a short α-helix, a feature common to PDZ accessory tails and CaMBD (Lee and Zheng, 2010; Figure 1). The segment is also predicted to form a hydrophobic cluster, reinforcing its role as a mediator of CaM/INAD protein-protein interactions. Similar to what was observed for dCRY, this region is conserved among Arthropoda, suggesting that the CaM/INAD interaction may be a common mechanism in this phylum (Figure 1 and Supplementary Figure S5).

A synthetic INAD peptide (residues 230–243) comprising the TMAKINKR motif was tested for binding to ^15^N-labeled CaM using ^15^N-HSQC experiments. This motif does not correspond to any prototypical CaM binding sequence (Rhoads and Friedberg, 1997; Hoeflich and Ikura, 2002). Nevertheless, the two residues Met-235 and Ile-238 could serve as hydrophobic anchors, defining a less characterized 1–4 motif already described for the CaM-binding domains of tobacco MAPK phosphatase (NtMPK1; Rainaldi et al., 2007) and the HIV-1 Matrix Protein (Samal et al., 2011). During the titration of ^15^N-CaM with INAD_230–243_, an incremental change in the position of many ^15^N-HSQC cross peaks was observed in response to peptide addition (Figure 4C), indicating a fast exchange regime. In this case, the assignment of the peptide bound state could be obtained by simply tracking the incremental movement of the perturbed peaks. A more detailed analysis shows that during the titration a number of peaks, characterized by a relatively large shift, become broader and then sharpen again, giving the titration curve a sigmoidal appearance (Supplementary Figure S6). Both features indicate a fast-to-intermediate exchange regime for some of the peaks. In these conditions, fitting the titration curves would be largely inaccurate. Altogether, this behavior is associated with a K_d_ in the μM range. Although the spectra show that several residues distributed in the entire protein are perturbed during titration, the largest shifts involve peaks corresponding to residues of the C-terminal CaM lobe. The overall chemical shift change was 2.5 and 4.5 ppm for the N- and C-terminal lobe, respectively, suggesting a preferential interaction of the peptide with this domain. Specifically, the most significant chemical shift changes in ^1^H-^15^N resonances (at least one standard deviation above average) were observed for the following residues: Val-55, Lys-77, Asp-78, Glu-83, Asp-93, Val108, Met-109, Thr-110, Asn-111, Gly-113, Lys-115, Ala-128, Gln-143, Met-144, Thr-146 and Lys-148 (Figure 4D). Most of these residues cluster on a hydrophobic surface containing four methionines, which becomes exposed only in Ca^2+^-loaded CaM.

Chemical shifts based 3D structure prediction, although limited in quality by the sole ^1^H-^15^N spectra, suggests CaM to assume a compact conformation resembling that of CaM bound to AKAP79 CaM binding domain (Patel et al., 2017), with both C-lobe and N-lobe participating in the interaction with INAD_230–243_ (Supplementary Figure S7). The C-lobe, in particular, is predicted to assume a more open conformation supporting the preferential interaction presumed for this domain. Of note, a similar prediction using the chemical shifts measured in a 1:2 CaM/INAD_230–243_ solution, suggests that, in these conditions, the N-lobe also goes through a conformational change to a more open conformation, possibly engaging an additional interaction with a second peptide (Supplementary Figure S7). The overall quality of this model, however, is almost modest and does not allow further speculations on binding geometry.

**Scheme 3:**
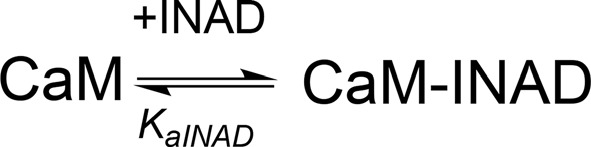
Model describing the binding of a single INAD molecule to CaM protein, in the presence of Ca^2+^.

ITC experiments showed that, in the presence of 5 mM Ca^2+^, CaM interacts with INAD with an exothermic reaction, as indicated by the negative peaks following each injections of the peptide into the protein solution (Supplementary Figure S8A). A single binding event is inferred from the integrated heat data (Figure 3B and Supplementary Figure S8B). Due to the weak binding of INAD to CaM, the sigmoidicity of the curve was not optimal to obtain a reliable stoichiometry value, as no inflection point is present in the binding isotherm to provide this information. Therefore, restrictions in the binding parameters had to be made to obtain a reliable data analysis, by fixing the stoichiometry and r_M_ to one, implying therefore a single binding site for INAD to CaM (Scheme 3).

A fit (GoF = 72.83%) using a single site model indicates that one INAD molecule binds CaM with LA *K_aINAD_* = 6.08 ± 0.06 × 10^3^ M^−1^, *K*_d1INAD_ = 164 ± 2 μM, with a favorable enthalpic contribution (*ΔH* = −8.52 ± 0.01 kcal mol^−1^) and a negative entropy change (*ΔS* = −11.3 cal mol^−1^ K^−1^).

### Competition Between INAD and dCRY for CaM

**Scheme 4:**
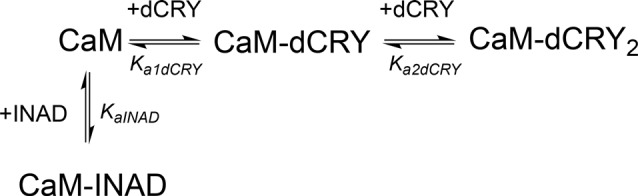
Model describing the competition of INAD and dCRY for the HA site of CaM and the subsequent binding of dCRY to the LA site in the CaM-dCRY complex.

In order to understand whether the site occupied by INAD corresponds to the HA or LA site observed for dCRY, a competition experiment was carried out by titrating INAD over a CaM solution, preincubated with a 1:1 concentration of dCRY. In these conditions, we expect that only the HA site is occupied by dCRY because of the large difference in affinity between the HA and LA sites. The reaction was monitored using ITC. The absence of significant heat of reaction (Supplementary Figure S9A) indicated that dCRY binding to the HA site prevents INAD from binding to the protein, suggesting that dCRY and INAD compete for the same site. The INAD peptide is not able to displace dCRY because the affinity of INAD for CaM is four orders of magnitude lower than that of dCRY (K_d_ 164 μM vs. 58 nM). On the other hand, when a dCRY solution is titrated over CaM preincubated with a four times excess of INAD, exothermic peaks are observed following each injection (Supplementary Figure S9B). The shape of the integrated curve (Figure 3C and Supplementary Figure S9C), confirms that dCRY is able to outcompete INAD for binding to the protein into the HA site, before occupying the LA site. A fit of the integrated heat data, however, is quite complex, as the binding curve represents the sum of the contributions of the multiple binding isotherms deriving from the different equilibria involved. These include the binding of dCRY to CaM in the HA site and in the LA site, and the concomitant dissociation of INAD from the HA site, as well as the competition between the different species. Therefore, we used a global fit analysis approach, available in the AFFINImeter software, to combine all the information comprised in the three binding isotherms representing the single equilibria and the competition experiment (Figures 3A–C). Using the model builder tool of AFFINImeter, we first constructed a binding scheme describing the competition of INAD and dCRY for the HA site of CaM, and the subsequent binding of dCRY to the LA site to give the CaM-dCRY_2_ complex (Scheme 4).

**Scheme 5:**
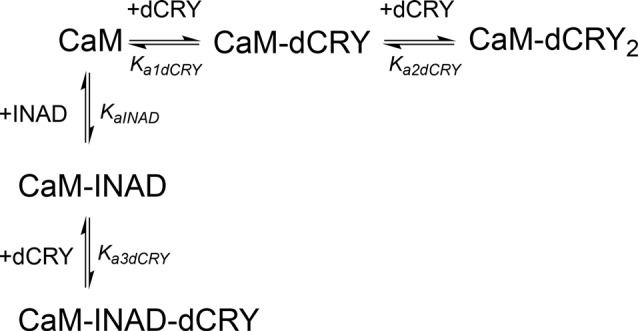
Model describing the competition of INAD and dCRY for the HA site of CaM and the subsequent binding of dCRY to the LA site, either in the CaM-dCRY or in the CaM-INAD complex.

The fitting of the data using this model (GoF = 33.83% for the competition curve) is clearly not satisfactory (Supplementary Figure S9C). Therefore, a different binding model (Scheme 5) was applied to include the formation of a complex in which dCRY binds the LA site in the CaM-INAD complex, giving a CaM-INAD-dCRY ternary complex. The binding affinity of dCRY to CaM-INAD was considered as an independent fitting parameter, to investigate whether the presence of either INAD or dCRY bound to the HA site could influence the interaction of dCRY with the LA site.

The fit deriving from this scheme was clearly more consistent (GoF = 64.7%) with the integrated data (Figure 3C). The derived affinity and dissociation constants are *K_a1dCRY_* = 1.04 ± 0.02 × 10^7^ M^−1^, *K*_d1dCRY_ = 96 ± 2 nM; *K*_a2dCRY_ = 1.02 ± 0.03 × 10^4^ M^−1^, *K*_d2dCRY_ = 98 ± 2 μM; *K*_a3dCRY_ = 5.6 ± 0.1 × 10^6^ M^−1^, *K*_d3dCRY_ = 179 ± 3 nM; *K*_aINAD_ = 9.95 ± 0.08 × 10^3^ M^−1^, *K*_dINAD_ = 100.5 ± 0.8 nM). The obtained enthalpy changes are *ΔH*_1dCRY_ = −3.963 ± 0.007 kcal mol^−1^, *ΔH*_2dCRY_ = −9.2 ± 0.1 kcal mol^−1^, *ΔH*_3dCRY_ = +1.10 ± 0.2 kcal mol^−1^ and *ΔH*_INAD_ = −4.132 ± 0.03 kcal mol^−1^, from which the corresponding values of entropies were calculated as *ΔS*_1dCRY_ = +18.8 cal mol^−1^ K^−1^, *ΔS*_2dCRY_ = −15.0 cal mol^−1^ K^−1^, *ΔS*_3dCRY_ = +34.6 cal mol^−1^ K^−1^, *ΔS*_INAD_ = +4.42 cal mol^−1^ K^−1^. The results of the global fit analysis, reported in Figure 3, suggest that binding of INAD to the HA site of CaM increases the affinity of dCRY for the LA site by two orders of magnitudes. This change in the affinity possibly involves a protein conformational change, reflected in the change of the enthalpy value from negative to positive. This event renders the formation of a CaM-INAD-dCRY ternary complex possible, as derived from the concentration distribution of the different species upon dCRY titration into the CaM-INAD solution (Figure 3D).

## Discussion

CaM signaling regulates visual responses in both vertebrates and invertebrates. Previous studies have shown that CaM interacts directly with INAD, the main scaffold of the phototransduction complex (Xu et al., 1998). CaM is also known to modulate heterodimerization of several proteins (Kilhoffer et al., 1992; Hoeflich and Ikura, 2002), acting as a strong regulator of physiological responses. Our previous work (Mazzotta et al., 2013) suggested that the interaction between dCRY and INAD might be modulated by CaM. Experimental validation by yeast two-hybrid and CoIP assays confirms that dCRY binds CaM, forming a ternary protein complex *in vivo*, together with INAD. Using an integrated approach, in the present study we also demonstrate that both single CaM domains bind dCRY. It has been already proposed that CaM may act as an adaptor or recruiter protein, easing the assembly of macromolecular complexes if each of the two CaM lobes is able to independently bind separate targets (Yamniuk et al., 2007). In the present study, we identified the dCRY portion interacting with CaM and we propose that it is located at the beginning of the flexible C-terminal tail containing the linear motifs which are important for PDZ domain recognition (Hemsley et al., 2007). To confirm this hypothesis, we titrated ^15^N-labeled CaM with a peptide encompassing dCRY residues 490–516 and showed that the addition of this peptide determined a shift and a concomitant broadening of several peaks in the HSQC spectrum of CaM. This behavior indicates that the selected dCRY portion binds CaM with low micromolar affinity. Calorimetric measurements also support this interpretation. Our results have narrowed down the previously identified INAD CaM binding domain to the α-helical motif (MAKI, aa residues 235–238) upstream from the PDZ2 domain. This sequence has previously been observed to be necessary for the interaction with dCRY (Mazzotta et al., 2013).

The typical CaM binding domain consist of a short peptide (15–30 residues) rich in bulky hydrophobic residues and positive charges, with a propensity to fold into an α-helix. Initial attempts to classify CaM binding motifs have been carried out based on the spacing between anchor hydrophobic residues (Ishida and Vogel, 2006). It is now clear that peptide sequences able to bind CaM are difficult to categorize, as numerous unclassified binding sequences have been reported (Yap et al., 2000). Neither dCRY nor INAD contain a motif corresponding to any of the known classical CaM-binding site patterns. In the present study, we identified INAD residues 233–240 as a putative CaM-binding site, in a region of INAD previously shown to be involved in CaM binding (Xu et al., 1998). This region has a strong propensity to fold into an α-helix and contains two hydrophobic residues. In the helical conformation, these residues localize on the same side of the structure, in the orientation expected for them to act as anchor points. The presence of positive charges completes the typical CaM binding motif requirements. Interestingly, the relative position of these residues resembles that of the CaMBD region of *Nicotiana tabacum* MAPK phosphatase-1 (NtMKP1; Yamakawa et al., 2004). The motif responsible for NtMKP1 binding to CaM defines a novel, unconventional, 1–4 motif in which two anchor residues are separated by only two amino acids, one of which is basic (Yamakawa et al., 2004).

In our NMR experiments, the addition of INAD_230–243–_ to ^15^N-labeled CaM caused incremental chemical shift changes in the HSQC spectrum, indicating fast-to-intermediate exchange between the free and bound states, consistent with low μM affinity. The largest shifts were observed for residues in the C-terminal lobe of CaM, suggesting a preferential interaction with this domain. Calculation of the CaM 3D structure from ^1^H-^15^N chemical shifts predicts that the protein binds INAD in a “wrap around” mode with stoichiometry 1:1. In this condition, the C-lobe in particular is predicted to assume an open conformation that presumably triggers the interaction, where the N-lobe maintains a closed-like conformation. Remarkably, a peptide of similar length derived from NtMPK1 was shown to bind to the C-terminus of soybean CaM with HA and subsequently, at higher peptide concentrations, to the N-terminal lobe (Rainaldi et al., 2007). A similar behavior was later observed also for a short peptide spanning residues 11–28 of HIV-1 matrix protein (MA_11–28_), which binds specifically to the C-terminal domain of CaM (Samal et al., 2011). This peptide also presents a 1–4 motif similar to INAD_230–243_ and to the NtMPK1 peptide. In our study, the yeast two-hybrid assays confirmed that the separate CaM domains effectively bind INAD in an independent manner, with efficiency comparable with full length CaM. The analysis of spectral changes during the NMR titrations clearly shows that both peptides interact with CaM in the affinity range expected for weak transient interactions, as those mediated by linear motifs described here (Perkins et al., 2010). Collectively, the data presented here support a role for CaM in facilitating the assembly of a macromolecular complex between dCRY and INAD.

**Figure 5:**
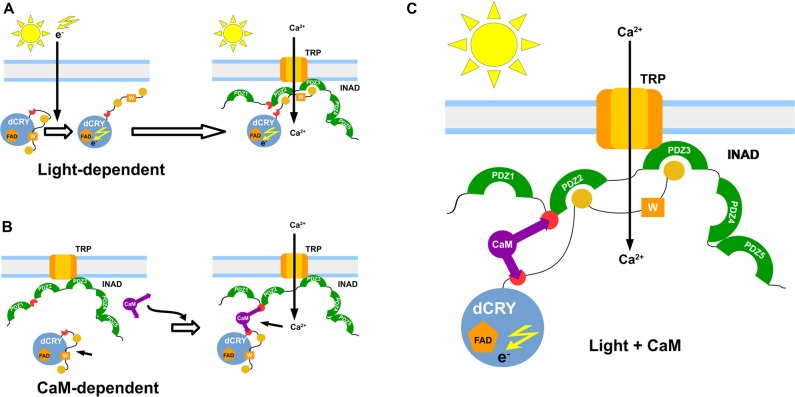
Functional model of the interaction. Graphical representation of the proposed dCRY/INAD interaction mechanisms. The blue sphere represents dCRY, while INAD is represented in green. The purple item represents CaM. The light-dependent induction is presented in **(A)**, with photons promoting a conformational change of dCRY, thus allowing interaction with INAD. A similar interaction is also mediated by CaM **(B)**, which forms a ternary protein complex with dCRY and INAD. Both mechanisms are proposed to cooperate in strengthening signal transmission **(C)**.

We have also observed that, while occurring both in light and dark, the INAD interaction with CaM is significantly stronger in the presence of light. Even though light was not expected to influence the binding activity, our result could be explained taking into consideration the reported effects of light on a non-photosynthetic organism such as *Saccharomyces cerevisiae*. Exposure to continuous blue light activates a yeast stress-response pathway that leads to an increase in intracellular Ca^2+^ levels (Bodvard et al., 2013). In turn, this modulates the nuclear localization dynamics of stress-regulated transcription factors, ultimately triggering a light-induced gene expression response (Bodvard et al., 2013). The observed effect of light on the CaM affinity to dCRY was somehow expected due to light regulation on dCRY (Rosato et al., 2001; Hemsley et al., 2007; Mazzotta et al., 2013). Considering the above mentioned yeast response to blue light (Bodvard et al., 2013), it can be hypothesized that the photo-induced dCRY conformational change is modulated by CaM signaling. Although not all the available evidence supports this hypothesis (Ozturk et al., 2011, 2014; Vaidya et al., 2013; Masiero et al., 2014), the involvement of Ca^2+^ dependent signaling pathways in fly circadian timekeeping is well established. Intracellular Ca^2+^ buffering in pacemaker neurons results in a dose-dependent period lengthening of free-running behavioral rhythms, mirrored by a slower accumulation of PAR domain protein 1 (PDP1), a crucial component of the interconnected transcriptional/translational *Drosophila* feedback loops (Harrisingh et al., 2007). Recently, an endogenous daily rhythm in intracellular Ca^2+^ has been detected in pacemaker neurons, which varies as a function of the time of day (Liang et al., 2016). A strong correlation was observed in the phase relationship between the peak of Ca^2+^ rhythms and the daily peaks of locomotor activity, for both morning (M) and evening (E) oscillators (Grima et al., 2004; Liang et al., 2016). Our *in vivo* experiments show that CaM is part of the complex formed by dCRY and INAD in fly photoreceptors and this association depends on Ca^2+^. This establishes a connection between CaM signaling and circadian clocks in photoreceptors, with the link being dCRY. The Ca^2+^ oscillation observed in pacemaker neurons was found to be independent from dCRY (Liang et al., 2016). However, dCRY plays different functions. In circadian clock neurons, dCRY acts as a circadian photopigment contributing to the resetting of the molecular clock by promoting light-dependent TIM degradation (Yoshii et al., 2016); in contrast, in peripheral tissues, including the compound eyes, it has been suggested that it might provide an integral component of the molecular clock (Ivanchenko et al., 2001; Krishnan et al., 2001; Collins et al., 2006). Moreover, in compound eyes it is also associated with the cytoplasmic membrane and contributes to the modulation of visual sensitivity (Mazzotta et al., 2013). Therefore, we hypothesize an involvement of dCRY in a Ca^2+^ dependent signaling mechanism, impinging on the circadian fly timekeeping. To put our findings into context, we propose a model in which light and CaM co-modulate the phototransduction cascade (Figure 5). A fast light response stimulates dCRY conformational changes, which in turn allow a prompt interaction with INAD when the fly is exposed to blue light. An additional slower mechanism, regulated by CaM, consolidates the initial light-induced response and this synergistic action strengthens the physiological output. Our NMR and calorimetric data supporting low μM affinities for the interactions of CaM with dCRY and INAD perfectly fit this scenario. Within this setting, CaM would act as an adaptor protein, stabilizing the interaction between dCRY and INAD enhancing their functional responses. This is in line with the notions that the ability to readily form and break interactions allows fast responses to cellular perturbations and changes in the environment (Stein et al., 2009) and that the simultaneous presence of multiple weak interactions enhances the stability of the complex and allows a more deterministic regulation of cell processes.

## Author Contributions

GMM, MB, GM, SM, RC and ST designed research. GMM, MB, GM, MD, PC, SA, BZ and MS performed research. GMM, MB and GM analyzed data. GMM, MB, GM, SM, BZ, RC and ST wrote the article.

## Conflict of Interest Statement

The authors declare that the research was conducted in the absence of any commercial or financial relationships that could be construed as a potential conflict of interest.

## Footnotes

^1^http://www.bmrb.wisc.edu

^2^https://www.affinimeter.com
