# Supplementary figures and images for: Calmodulin Enhances Cryptochrome Binding to INAD in Drosophila Photoreceptors
Source: Front Mol Neurosci. 2018 Aug 20;11:280. doi: 10.3389/fnmol.2018.00280 (PMC6109769; doi:10.3389/fnmol.2018.00280)

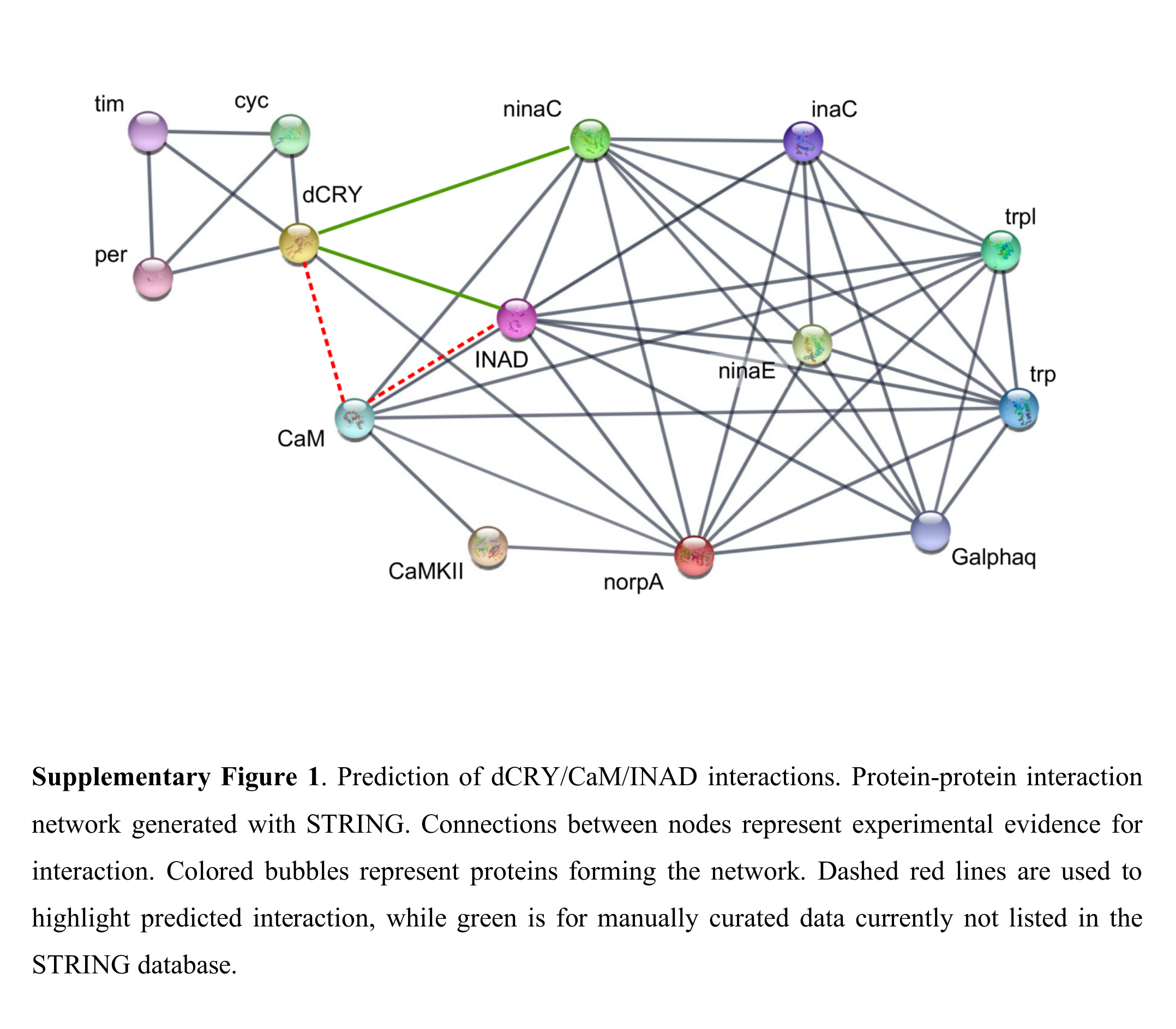

Supplement: Supplementary file 1 [file Image_1.TIFF]

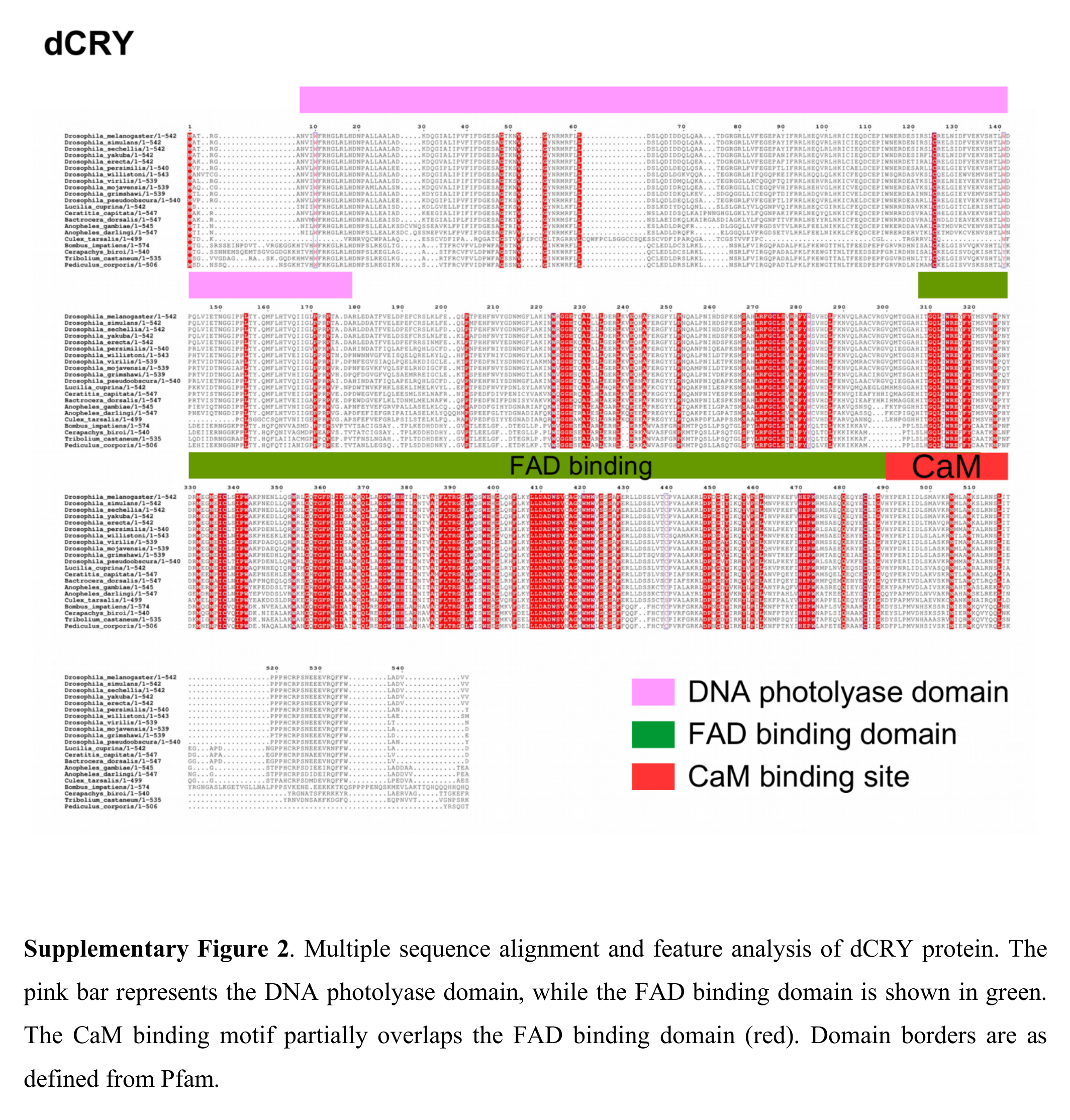

Supplement: Supplementary file 2 [file Image_2.TIFF]

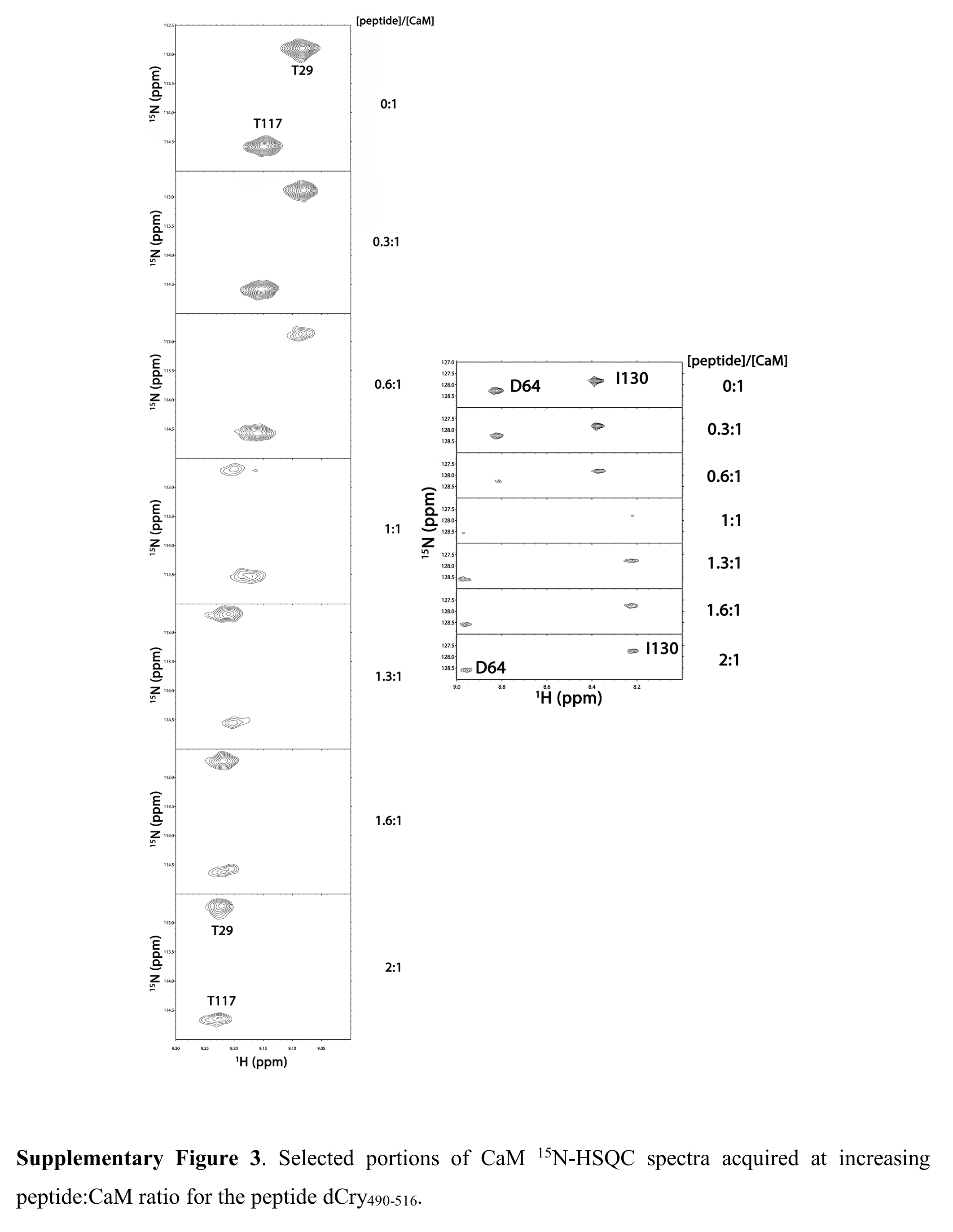

Supplement: Supplementary file 3 [file Image_3.TIFF]

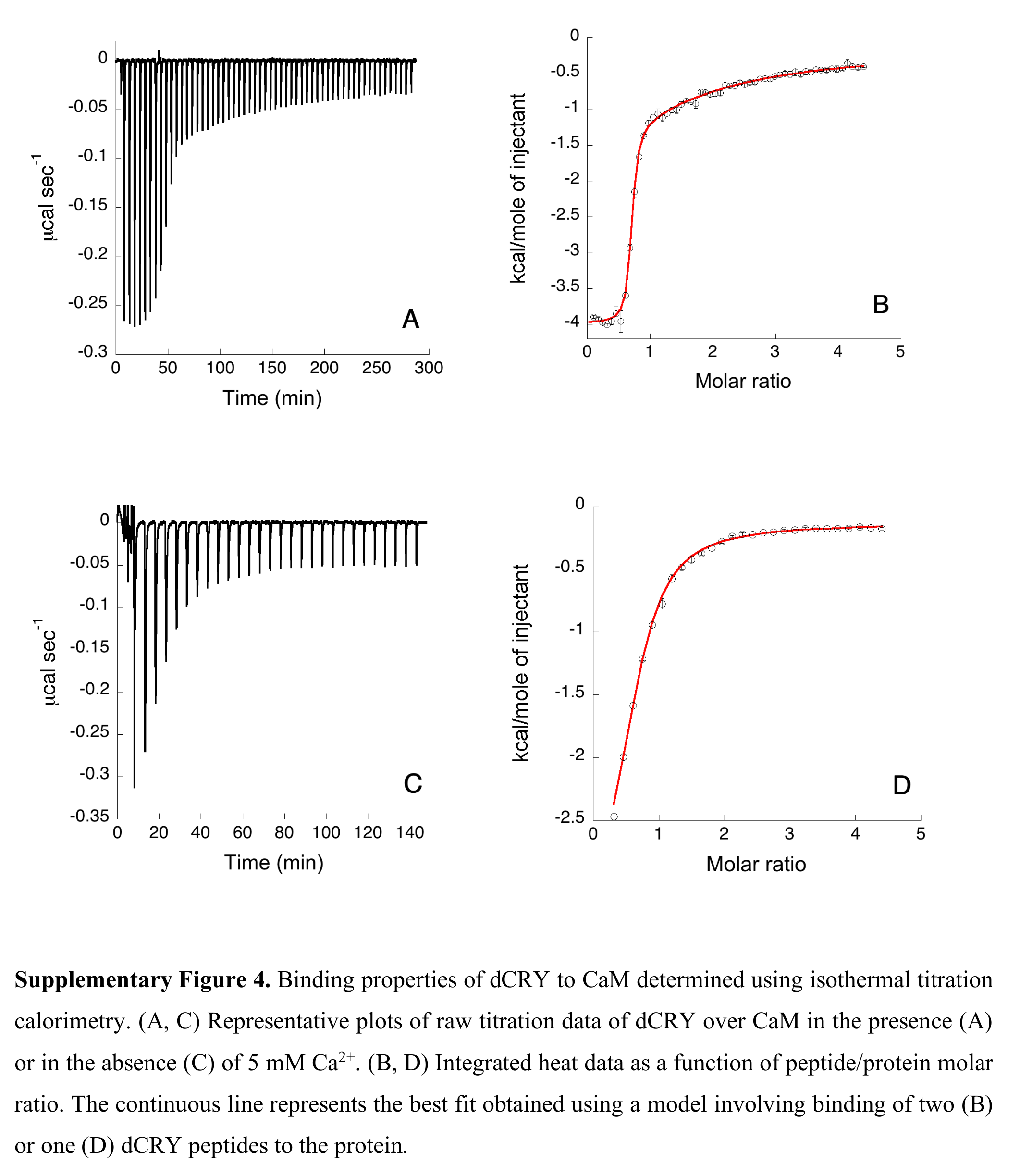

Supplement: Supplementary file 4 [file Image_4.TIFF]

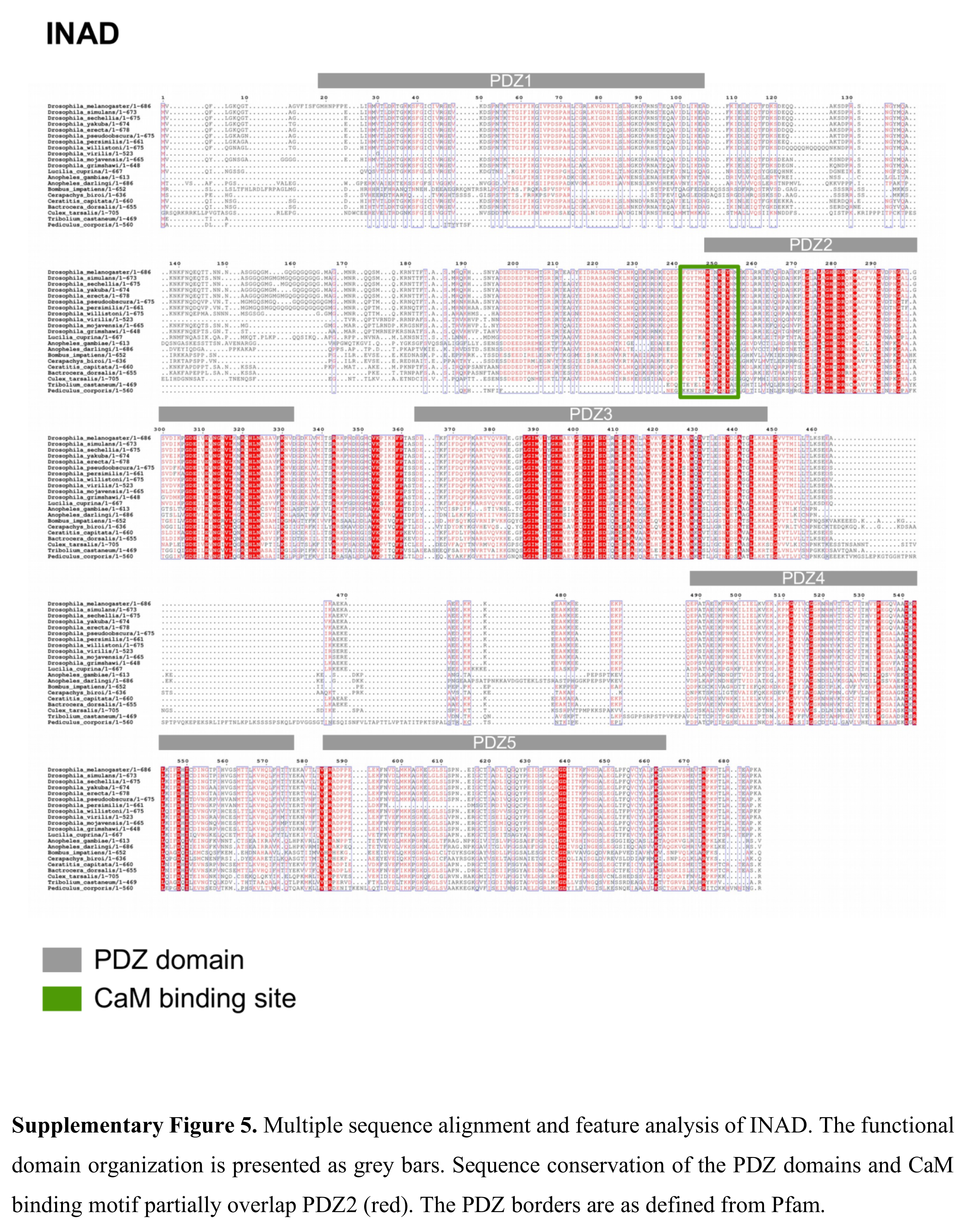

Supplement: Supplementary file 5 [file Image_5.TIFF]

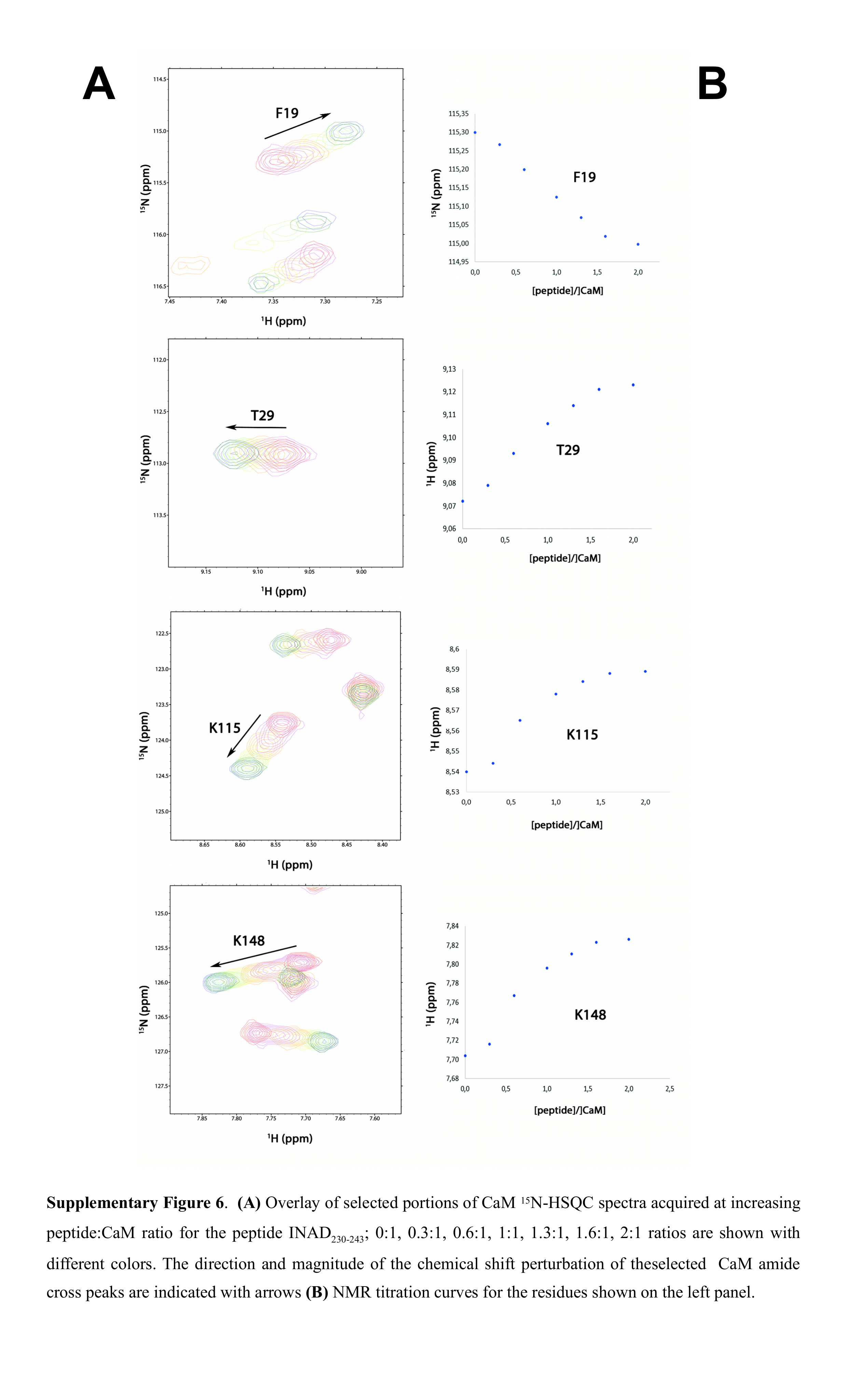

Supplement: Supplementary file 6 [file Image_6.tiff]

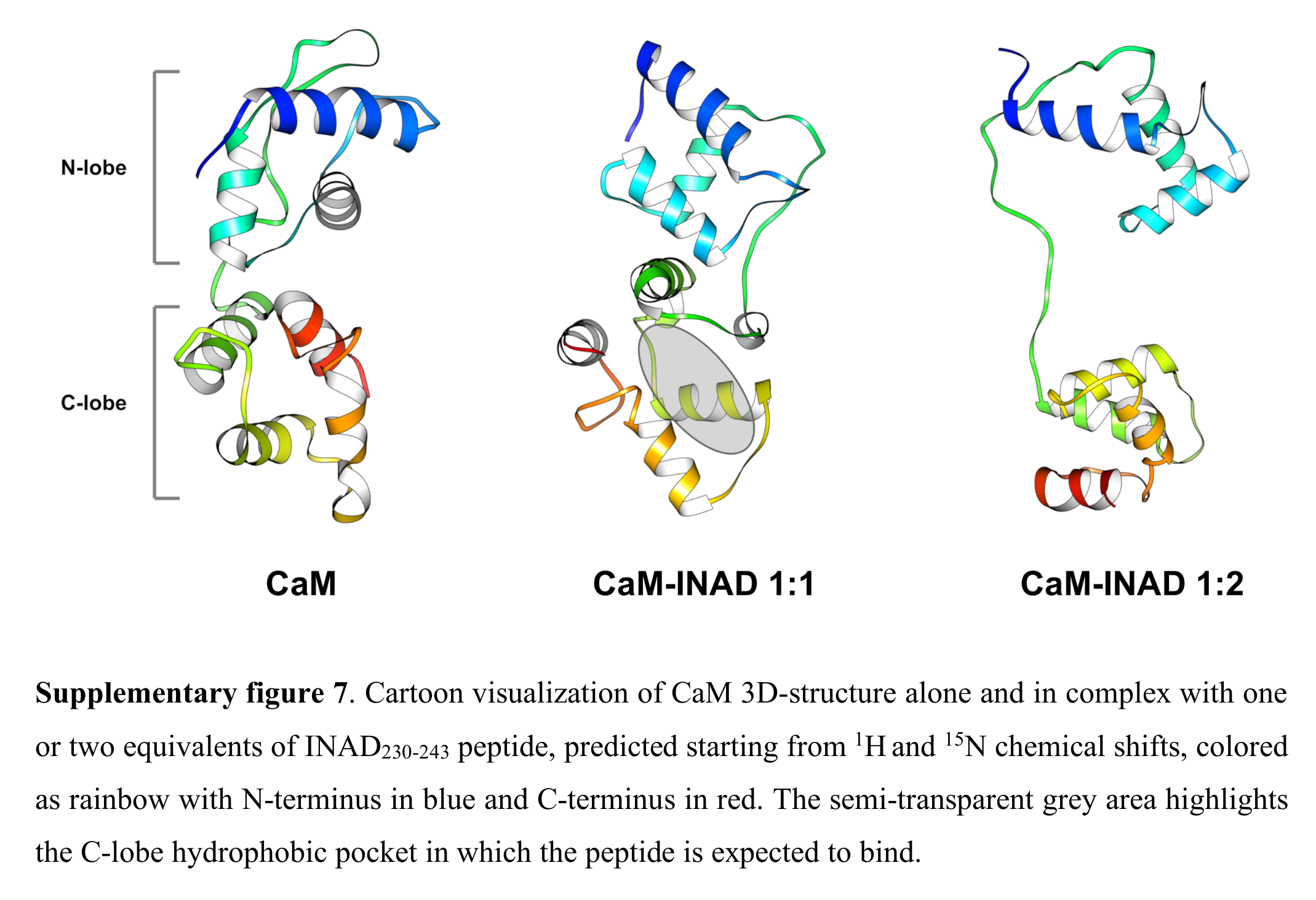

Supplement: Supplementary file 7 [file Image_7.TIFF]

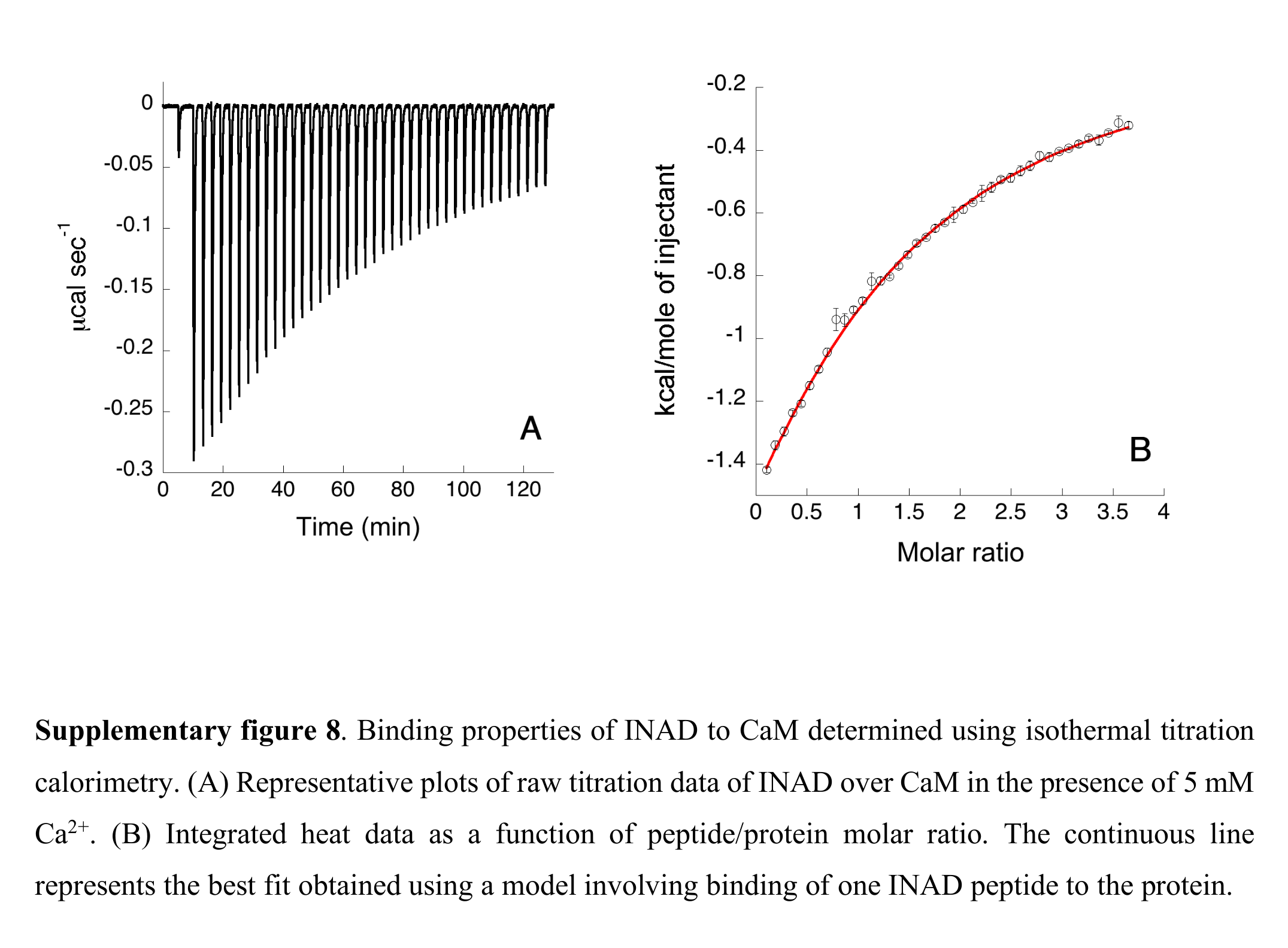

Supplement: Supplementary file 8 [file Image_8.TIFF]

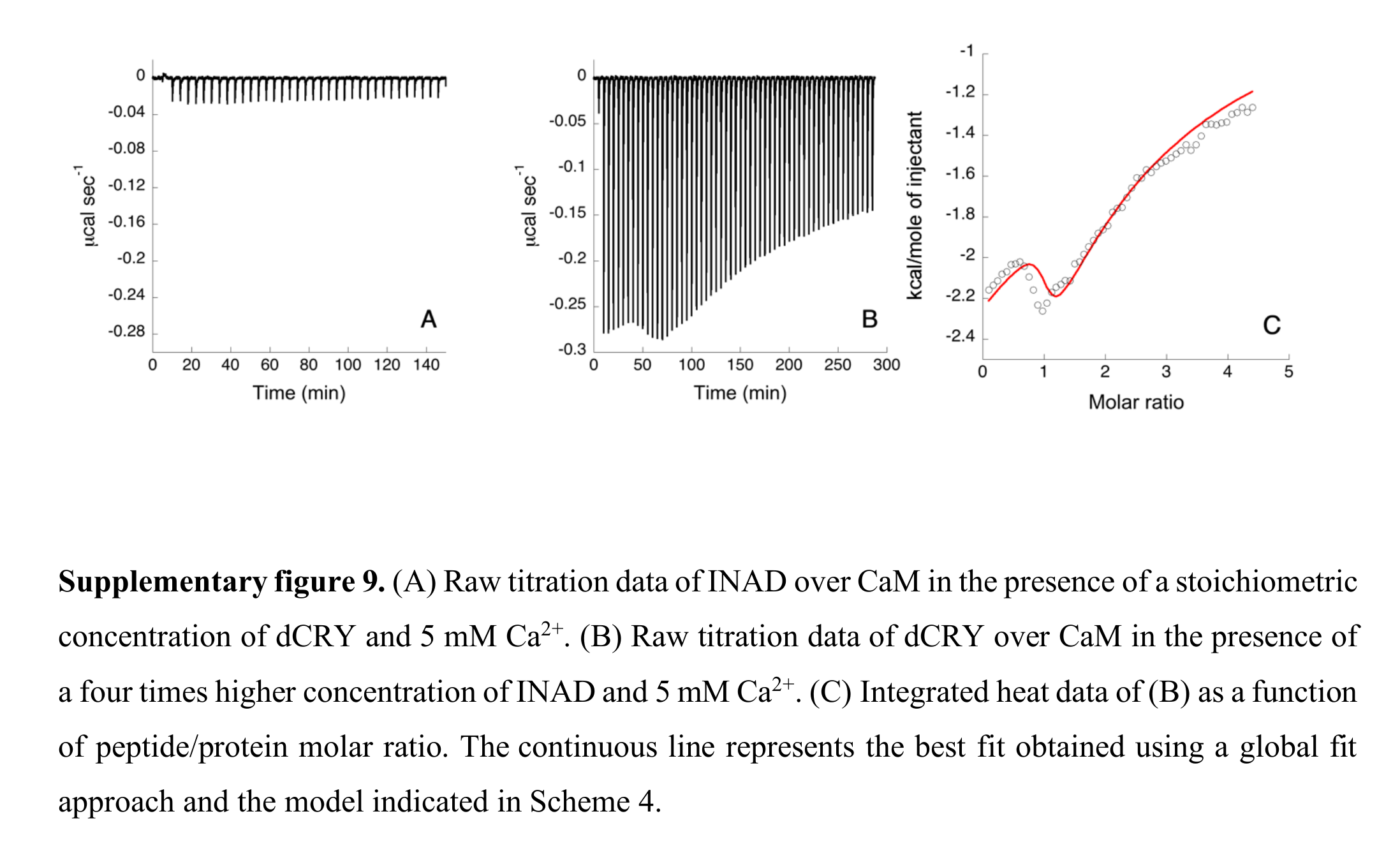

Supplement: Supplementary file 9 [file Image_9.TIFF]
