# Supplementary material for: Calmodulin Enhances Cryptochrome Binding to INAD in Drosophila Photoreceptors
Source: Front Mol Neurosci. 2018 Aug 20;11:280. doi: 10.3389/fnmol.2018.00280 (PMC6109769; doi:10.3389/fnmol.2018.00280)
Supplement: Supplementary file 10 [file Table_1.DOCX]

| PRIMER | SEQUENCE (5’-3’) |
| --- | --- |
| pEG_Calm_F | *cggcgactggctggaattc*ATGGCTGACCAACTGACAGAAG |
| pEG_Calm_R | gctgcaggtcgactcgagTCACTTTGCTGTCATCATTTGTAC |
| pEG_Calm_NR | *gctgcaggtcgactcgag*TCAGTCTGTGTCTTTCATTTTTCTAGC |
| pEG_Calm_CF | *cggcgactggctggaattc*ATGAAAGACACAGACAGCGAAGAAG |
| pJG_Calm_F | *gtgccagattatgcctctcccgaattc*ATGGCTGACCAACTGACAGAAG |
| pJG_Calm_R | cgaagaagtccaaagcttctcgagcatatgTCACTTTGCTGTCATCATTTGTAC |
| pJG_Calm_NR | cgaagaagtccaaagcttctcgagcatatgTCAGTCTGTGTCTTTCATTTTTCTAGC |
| pJG_Calm_CF | *gtgccagattatgcctctcccgaattc*ATGAAAGACACAGACAGCGAAGAAG |

XlCALM2: NM_001087395

**Supplementary Table 1. Nucleotide sequence of primers used.**
